# Evaluating the impact of the weather conditions on the influenza propagation

**DOI:** 10.1186/s12879-020-04977-w

**Published:** 2020-04-05

**Authors:** David E. Singh, Maria-Cristina Marinescu, Jesus Carretero, Concepcion Delgado-Sanz, Diana Gomez-Barroso, Amparo Larrauri

**Affiliations:** 1 Carlos III University of Madrid, Leganés, Spain; 2 Barcelona Supercomputing Center, Barcelona, Spain; 3 CIBER en Epidemiología y Salud Pública (CIBERESP), Madrid, Spain; 4 National Centre for Epidemiology, Carlos III Institute of Health, Madrid, Spain

**Keywords:** Influenza epidemic, Simulation, Meteorological model

## Abstract

**Background:**

Predicting the details of how an epidemic evolves is highly valuable as health institutions need to better plan towards limiting the infection propagation effects and optimizing their prediction and response capabilities. Simulation is a cost- and time-effective way of predicting the evolution of the infection as the joint influence of many different factors: interaction patterns, personal characteristics, travel patterns, meteorological conditions, previous vaccination, etc. The work presented in this paper extends EpiGraph, our influenza epidemic simulator, by introducing a meteorological model as a modular component that interacts with the rest of EpiGraph’s modules to refine our previous simulation results. Our goal is to estimate the effects of changes in temperature and relative humidity on the patterns of epidemic influenza based on data provided by the Spanish Influenza Sentinel Surveillance System (SISSS) and the Spanish Meteorological Agency (AEMET).

**Methods:**

Our meteorological model is based on the regression model developed by AB and JS, and it is tuned with influenza surveillance data obtained from SISSS. After pre-processing this data to clean it and reconstruct missing samples, we obtain new values for the reproduction number of each urban region in Spain, every 10 minutes during 2011. We simulate the propagation of the influenza by setting the date of the epidemic onset and the initial influenza-illness rates for each urban region.

**Results:**

We show that the simulation results have the same propagation shape as the weekly influenza rates as recorded by SISSS. We perform experiments for a realistic scenario based on actual meteorological data from 2010-2011, and for synthetic values assumed under simplified predicted climate change conditions. Results show that a diminishing relative humidity of 10% produces an increment of about 1.6% in the final infection rate. The effect of temperature changes on the infection spread is also noticeable, with a decrease of 1.1% per extra degree.Conclusions: Using a tool like ours could help predict the shape of developing epidemics and its peaks, and would permit to quickly run scenarios to determine the evolution of the epidemic under different conditions. We make EpiGraph source code and epidemic data publicly available.

## Background

Seasonal influenza may not make headlines, but together with pneumonia, it is one of the top ten causes of death worldwide. Influenza epidemics results in 3 to 5 million cases of severe illness a year, which puts a high burden on health providers and results in loss of productivity and absenteeism, such as mentioned by the World Health Organization in [1]. It‘s been long known that in temperate climates these seasonal epidemics occur mostly in winter, and typical hypotheses assigned the blame to people being in closer proximity for longer periods of time, or lowered immune systems. In general, meteorological conditions affect virus transmission due to multiple effects: virus survival rates, host contact rates and immunity, and the transmission environment (except the case of direct or short-range contact). While these factors may have an influence, the solid evidence sustains the hypothesis that the virus‘s best surviving conditions are low temperatures and low absolute humidity. One of the goals of the current research in this field is to understand this relationship to be able to develop a more accurate seasonal influenza model for both temperate and tropical regions. As a motivation of this work, JT et al. [2] conclude that environment factors may become more important for a future predictive model of the effects of climate change. In a previous paper [3], some of the authors of this paper studied the interaction of the spatio-temporal distribution of influenza in Spain and the meteorological conditions during five consecutive influenza seasons. The work uses real influenza and meteorological data in combination with statistical models to show that there is a relationship between the transmission of influenza and meteorological variables like absolute humidity and amount of rainfall. In this work we use the same data sources (SISSS and AEMET agencies) following a different approach: we study some of these relationships from a simulation perspective, considering not only the existing influenza distributions but also the ones related to the climate change.

In this work we extend EpiGraph [4], an influenza simulator, with a meteorological model (MM) starting from the model developed by AB and JS [5]. In their paper AB and JS analyze monthly weather and influenza mortality data collected between 1973 and 2002 throughout all of the 359 US urban counties. Using a regression model, they conclude that there exist correlations between both absolute humidity and temperature with mortality. They report a quantitative assessment of the relation between mean daily humidity and temperature levels and mortality rates in different ranges. This is an extensive study and, as a result, we start from the assumption that their results are solid and appropriate to incorporate to EpiGraph in order to produce meteorological-dependent simulations based on real data. In this work we extend EpiGraph [4], an influenza simulator, with a meteorological model (MM) starting from the model developed by AB and JS [5]. In their paper AB and JS analyze monthly weather and influenza mortality data collected between 1973 and 2002 throughout all of the 359 US urban counties. Using a regression model, they conclude that there exist correlations between both absolute humidity and temperature with mortality. They report a quantitative assessment of the relation between mean daily humidity and temperature levels and mortality rates in different ranges. This is an extensive study and, as a result, we start from the assumption that their results are solid and appropriate to incorporate to EpiGraph in order to produce meteorological-dependent simulations based on real data.

Regarding other influenza simulators that consider weather conditions, PS et al. presents an agent-based simulation model [6] that evaluates the seasonal effects on the influenza propagation. Although the reproductive rates are generated synthetically without considering actual meteorological data, this paper shows, in a similar way than our work, the impact of changing reproductive rates on the course of the influeza pandemic. In the article [7], JS et al. simulate influenza transmission via a SIRS model modulated by climate data to obtain the basic reproduction number *R*_0_. Both JS et al. [8] and ACL et al. [9, 10] study the effects of humidity on influenza transmission from the point of view of virus survival and conclude that aerosol transmission is most efficient in low humidity conditions. ACL et al. [9, 10] and BX et al. [11] also conclude that aerosol transmission is more efficient at low temperatures. JS et al. [8] and JM et al. [12] also deduce that virus survival increases with decreasing the humidity values.

**Fig. 1:**
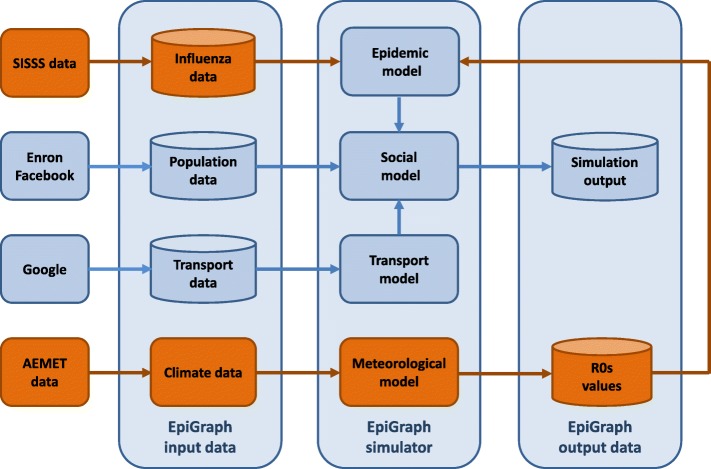
Overview of the data sources, processed data, and EpiGraph components

Epigraph simulations use real data for modelling the population, the spatio-temporal distribution of influenza, and the meteorological conditions. This simulator consists of different components and data sources shown in Fig. 1. The previous and novel components are represented in blue and orange colors, respectively. The simulator uses input data that is obtained from different sources including: (1) the influenza data, that contains information about the initial individuals that are infected; (2) the population data, that describes the individual interactions with others; (3) the transport data, that contains information about the movement of individuals between different locations and (4) the climate data, that contains the meteorological conditions existing during the simulated time span. This data feeds the different models implemented in the simulator. We briefly describe the three models that have been previously developed and presented in [4, 13].

The Epidemic model considers the propagation model of influenza extending the SIR (as explained in [14] by FB et al.) to include states for latent, asymptomatic, dead and hospitalized. The infective period has different phases which may affect the dissemination characteristics of the influenza virus as AME et al. describe in [15]. Each individual has a slightly different length for each infection state. We adopt most of the concrete values for the model parameters from the existing literature on flu epidemics (see [14–17]). You can find them all in [18].

The transport component models the daily commute of individual to neighboring cities (inter-city movement) and the long-distance travels for several days that represent commute of workers that need to reside at different locations or people that move at any distance for vacation purposes. The people mobility model is based on the gravity model proposed by CV et al.[19] that uses geographical information extracted from Google using the Google Distance Matrix API service.

The social model is an agent model that captures individual characteristics and specifies the interaction patterns based on existing interactions extracted from social networks. These patterns determine the close contacts of each individual during the simulation, which is a crucial element to model the spread of the infection. We extract interaction patterns from virtual interactions via email or social networks (Enron and Facebook) and scale them to approximate a physical connection of the whole network within an urban area. These connections are time-dependent to realistically capture the temporal nature of interactions, in our case modeled depending on the day of the week and time of day. The distribution of the population is in terms of four group types: school-age children and students, workers, stay-home parents, and retirees.

In this paper, as main contribution, we introduce a new component of the simulator (the meteorological model), that evaluates the impact of climate parameters on influenza propagation. This component is tuned with influenza surveillance data obtained from SISSS to provide realistic simulations. As far as we know, this work is the first simulator that integrate real meteorological data to predict the spatio-temporal distribution of influenza. We think that this contribution will help to better understanding the influenza propagation in real environments.

In the literature we can find different influenza simulators although none of the following consider meteorological factors in the simulation. Examples of them is the work of KK et al. [20] that presents an SIR-based epidemic simulator that permits to parametrize both the population characteristics and the epidemic process. The goal of this work is to identify the turning point (peak of the infected population) of the infection. Although the initial approaches for modelling the infection spreading across the contact network, our work consider a broader number of parameters and configuration of the network. HE et al. [21] analyze, by means of simulation, the relationship between social interaction patterns at workplaces and the virus transmission patterns during influenza pandemics. The main effort is geared towards the flexible specification of the different aspects involved in a simulation, such as intervention policies, social modelling, social organization of work, etc. SimFlu [22] is different from most epidemic simulators in that it focuses on the discovery of most probable future influenza variants starting from virus sequences published by the National Center for Biotechnology Information (NCBI). This work is complementary to the goal of most simulators, including ours, which is to understand and predict the spreading infection patterns of a known flu strand across a population. Their methodology is based on observing directional changes in subtypes of influenza over time.

JS and AK present a framework [23] to adjust an epidemic simulation based on real-time forecasts of infections from Google Flu Trends. The paper focuses on prediction of the timing of peak infection, but other metrics could be predicted as well. The authors of [24] simulate the spreading of influenza in an urban environment consisting of several close-by towns connected by trains. Their goal is to be able to model and simulate intervention policies.

Epiwork [25] was a European project in FP7 whose focus was to develop a tool framework for epidemic forecast. Within this project’s framework, WB et al. describe GLEaMviz [26], their tool for epidemic exploration which includes a simulator of transmission based on an accurate demographics of world’s population over which they superpose a (stochastic) mobility model. DB et al. [27] use human mobility extracted from airline flights and local commute (based on the gravity model) to predict the activity of the influenza virus based on Monte Carlo analysis. SM and SM [28] study the role of population heterogeneity and human mobility in the spread of pandemic influenza. In [29], the authors reconstruct contact and time-in-contact matrices from surveys and other socio-demographic data in Italy and use this matrix for simulation.

## Methods

### Climate data pre-processing

**Fig. 2:**
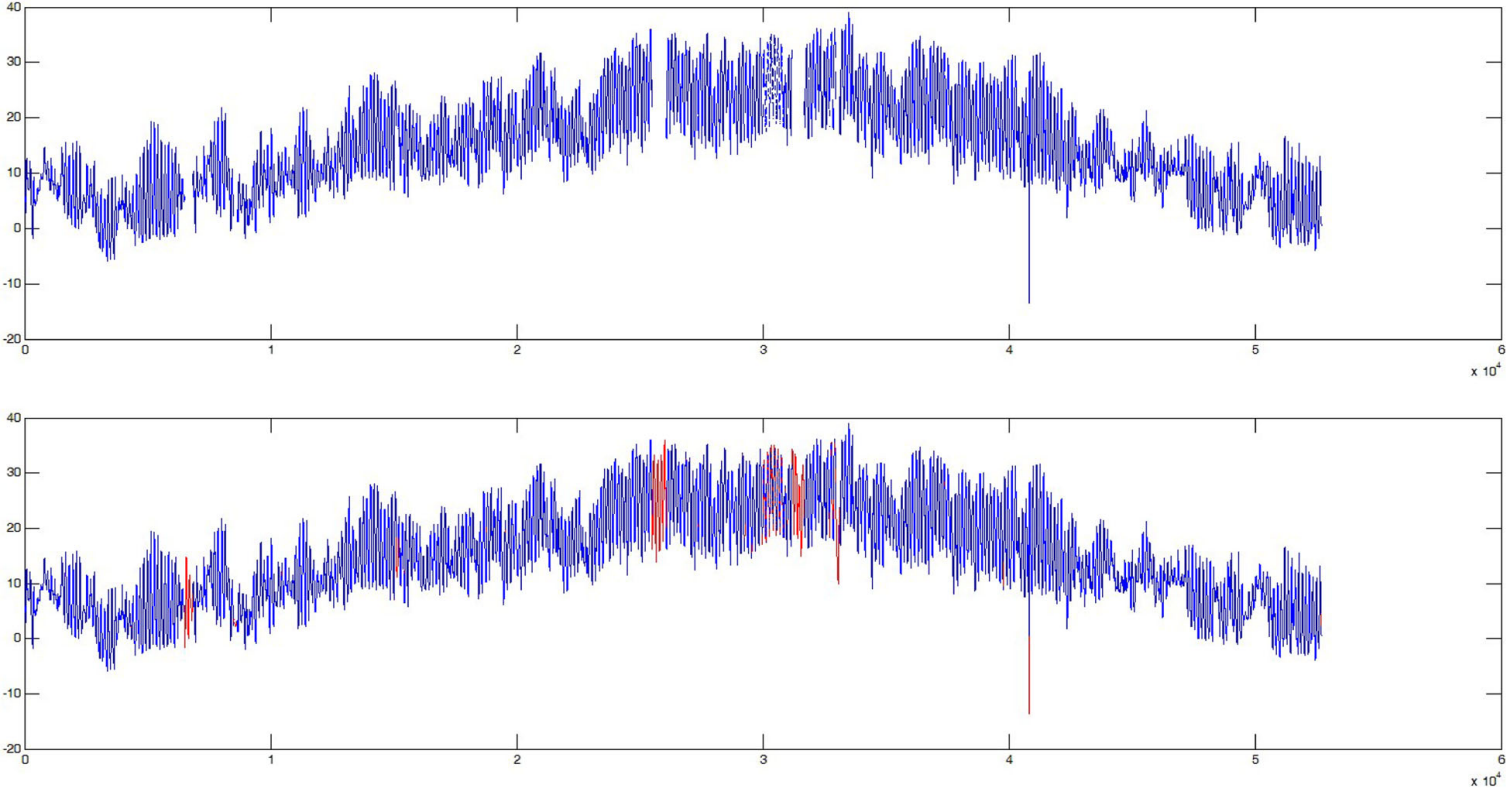
Data reconstruction example of temperature values for Alcobendas urban region (the reconstructed values are in red color). X-axis units conrresponds to tens of thousand samples and the total number of samples displayed is 52,560 (one sample every 10 minutes for one-year span)

Epigraph uses meteorological data provided by the Spanish Meteorological Agency (AEMET) to generate environment-dependent influenza simulations. The pre-processing stage is performed to obtain clean inputs for the meteorological model. First, the weather station nearest to each simulated urban region is identified. Our simulations consider 92 different urban regions with more than 100,000 inhabitants. In some cases, the station is within the city limits, while in others it is located in a nearby area (for instance at the region‘s airport). The data from each weather station is analyzed to reconstruct potentially missing samples. Sometimes it is the case that some station data samples are missing because the station was not operational during a given time period. These represents just a small fraction of the overall samples, but they have to be properly addressed. Figure 2 shows an example of how the original missing data (shown in upper figure) is reconstructed producing a complete samplingclearpage (reconstructed values are shown in the lower figure in red color). In order to add the missing samples, we have used the reconstruct data algorithm (missdata) included in the Matlab’s System Identification Toolbox. This toolbox permits the construction of mathematical models for dynamic systems, starting from measured input-output data.

The resulting data is then processed to filter non-realistic values. Some weather stations produce abnormal samples corresponding to non-realistic values that are too big or too small. Figure 2 shows an example of this kind of values around sample 41,000. We have corrected these cases with a Matlab algorithm we implemented to detect these peaks and correct them using an interpolation of the values from the previous days. These two steps are only performed once for each new meteorological input data and the results may be used for the rest of the process.

### Modeling the dependence of the infectious agent behavior on climate factors

This section describes how the R0s are obtained from the meteorological conditions. In addition to the notations introduced in the introduction, for the rest of the paper we will use *SH* for the specific humidity and $P^{*}_{H2O}$ for the equilibrium water vapor pressure. A related value to SH is Absolute Humidity (AH), which is the mass concentration that describes the amount of water vapour per volume of air. Previous studies [30, 31] suggest that AH (and by extension SH) are one of the main factors affecting the influenza virus transmission.

In EpiGraph we adopt the results of the regression model used by AB and JS [5]. In their 2012 paper, they analyze monthly weather and influenza mortality data collected between 1973 and 2002 throughout all the 359 US urban counties. Using regression, they conclude that there exists a strong correlation between absolute humidity and mortality, even when controlling for temperature, when the humidity drops below daily means of 6g/kg. Temperature correlations also exist, mainly in the daily ranges between -1.1C and 15.6C. In an earlier paper ([7]) JS et al. study the same dataset and simulate influenza transmission via a SIRS model modulated by the data to obtain the basic reproduction number *R*_0_. They also find best-fit parameter range combinations of *R*_0*max*_ between 2.6 and 4, and *R*_0*min*_ between 1.05 and 1.3. We adopt the pair of (*R*_0*max*_, *R*_0*min*_) that was found to be the best-fit parameter combinations they discover: *R*_0*max*_=3.52, *R*_0*min*_=1.12.

From the definition of the specific humidity (*SH*) and relative humidity (*RH*)—see RHP and DWG [32]—we know that:

1 $$ RH = SH * P / (0.622 + 0.378 * SH) * P^{*}_{H2O}  $$

We also know from Buck’s equation that the equilibrium water vapor pressure can be calculated using the formula:

2 $$  P_{H_{2}O} (T) = 0.61121* Exp((18.678-T/234.5)(T/(257.14+T)))  $$

where the temperature *T* is measured in degrees Celsius. This formula works best for values of T in the range of -80C to 50C. From known values *RH*, *P*, and *T*, and using Eqs. (1) and (2), we can calculate the specific humidity.

From laboratory experiments by JS et al. [7] we have:

3 $$ R_{0} = exp(a * q + b) + R_{0min} \simeq exp(a * SH + b) + R_{0min}  $$

where *a*=−180, *b*= log(*R*_0*max*_−*R*_0*min*_) and *q* is the 2-m above-ground specific humidity, which we approximate to *SH* at the given temperature. In this way, we obtain a value for $P^{*}_{H2O}$ in every sample (obtained every 10 minutes) using Eq. (2). From this value in combination with the values of *RH* and *P* we obtain the value of *SH* using Eq. (1). Finally, Eq. (3) computes the new *R*0 values for each urban region.

R0s are, therefore, time-dependent values that determine, in a stochastic process, how many susceptible individuals of an infected person’s connections could be potentially infected. This is the dynamic component of the infectivity of an individual with respect to the others. The other dynamic component is the stochastic transition between infective states [4], computed with variable probabilities.

Our model is not different for the different types / subtypes of influenza. The values of the model parameters (basic reproduction numbers for each stage of the disease) were chosen to fall in the ranges published by AB and JS, which are based on actual data for all types of influenza, over 30 years. We choose fixed *R*0s within the ranges, although this is a parameter that can be configured to vary. On the other hand, the evaluation was performed over data from the 2010-2011 influenza season over the whole territory of Spain, for all types of influenzas that were diagnosed. We consider that both the choice of *R*0 (based on exhaustive data) and the evaluation against real reported cases across Spain are comprehensive enough to validate our results.

### Simulator setup

#### Setting up the influenza model

The Spanish Influenza Sentinel Surveillance System (SISSS) comprises 17 networks of sentinel physicians (general practitioners and pediatricians) in 17 of the 19 Spanish regions, as well as the network-affiliated laboratories, including the National Influenza Reference Laboratory (National Centre for Microbiology, World Health Organization National Influenza Centre in Madrid). More than 800 sentinel physicians participated each season covering a population under surveillance of around one million—see [33, 34]. Sentinel physicians reported influenza-like illness (ILI) cases—integrating virological data collected in the same population—detected in their reference populations on a weekly basis, following a definition based on the EU-ILI, as described in [35]. For influenza surveillance, they systematically swab (nasal or nasopharyngeal) the first two ILI patients each week and sent the swabs to the network-affiliated laboratories for influenza virus detection. The information collected by the SISSS includes data on demographics, clinical and virological characteristics, seasonal vaccination status, chronic conditions, and pregnancy. Data is entered weekly by each regional sentinel network in a web-based application [36] and analyzed by the National Centre of Epidemiology to provide timely information on the evolving influenza activity in Spanish regions and at the national level. For example, during the 2011-2012 season, 651 sentinel physicians and 236 pediatricians participated to SISSS and surveyed a total population of 1,142,189, which represents 2.36% of the total population of Spain.

We obtained the SISSS data from the National Center of Epidemiology, Institute of Health Carlos III of Madrid (ISCIII). In order to produce realistic simulations, EpiGraph has to be properly configured. This configuration process consists of setting up two parameters: the date of the epidemic onset and the initial influenza-illness rates for each urban region.

The first parameter is the time of onset of the epidemics, which occurs during week 50 of 2010. At this time the national average incidence values for influenza are greater than 60 cases per 100,000 inhabitants, which is the threshold determined by SISS, based on data from the 2010-2011 seasonal epidemic, to be the start of the influenza season. In our simulation the exact date is the 13th of December of 2010.

The second parameter values were obtained from influenza surveillance data obtained from the SISSS corresponding to the influenza season 2010-11. From this data we obtained the reported weekly ILI rate at national and regional level in Spain. The data for the Murcia and Galicia communities are not available and we approximated them based on the data from the nearest community. These rates allow us to approximate the initial number of (clinically) influenza-like-infected individuals using the following formula, based on the study published online (in March 2017) in the Lancet Respiratory Medicine by ACH et al. [37].

4 $$  N_{tot} = \frac{N_{report}}{Symp*Attend}*F_{pos},  $$

where *N*_*report*_ are the cases that demanded medical attention, as reported by the SISSS, *F*_*pos*_ is the fraction of positive cases, *Symp* is the percentage of symptomatic individuals, and *Attend* is the percentage of those with symptoms that see a doctor. For instance, for the reported *N*_*report*_=90 cases per 100,000 inhabitants in week 50, and with values *F*_*pos*_=33*%* (empirical value for 2010-2011 in Spain), and *Symp*=23*%*,*Attend*=17*%* (values taken from the cited study), we calculate that the total number of infected individuals is of approximately 765 cases per 100,000 inhabitants - or 0.765% of the total population. We use this value to set up the initial conditions of the simulation (described in this section), but also to validate its results. Each community has a different *N*_*report*_, which leads to different numbers of initially infected individuals.

EpiGraph allows modeling at the level of each individual, and thus can simulate the effect of vaccination policies. To produce realistic results, we use different influenza vaccination coverages by age group; for those older than 65 we have used the vaccination ratios (per community) provided by the Ministry of Health, Social Services, and Equality of Spain. These values correspond to vaccination coverages collected by the National Health System [38]. For the rest of the population (individuals younger than 65) we have used the data provided by the Spanish Statistical Office, which is based on surveys done in each community. Given that the data are available at community level, we assume that all the urban areas located in the same community have the same vaccination coverages. Table 1 shows these percentages per community and age.

**Table 1:** Proportion of influenza vaccinated population by Spanish region

Age group	Andalucía	Aragón	Asturias	Balears	Cantabria	Castilla y León
16-64 years	21.8	20.4	23.8	18.0	22.5	27.7
>64 years	60.0	57.5	56.2	45.9	57.3	66.1
	C. Valenciana	Extremadura	Galicia	C. de Madrid	Murcia	Navarra
16-64 years	20.9	24.4	22.9	23.6	24.0	22.5
>64 years	50.6	50.8	52.4	58.2	49.3	60.1
	Castilla la Mancha	Cataluña	País Vasco	Rioja		
16-64 years	23.4	21.7	24.2	21.4		
>64 years	54.0	54.0	60.3	66.5		

As input of the mobility model, we use 85% workers and 15% students for short distance travel, and 50% workers, 30% students, 15% retired individuals, and 5% unemployed for long distance travel.

#### Calibrating the simulator

While EpiGraph accounts for many of the components that influence the spreading of the virus, the behavior of these parts and the values of the parameters (such as the initial infectious individuals or the vaccination rate) are unavoidably approximate. On their website, the World Health Organization reports that in annual influenza epidemics, 5-15% of the population are affected with upper respiratory tract infections [1]. We have therefore introduced a scaling factor which adjusts the infection propagation rate of each individual to produce, for each urban region, a final infection rate between 5% and 15% of the total population. These values are obtained in a pre-calibration phase of EpiGraph for the real climate conditions—performed only once— and are then used for all subsequent simulation experiments. Note that this is the only data –which is also based on real data– that we use for the calibration process. We do not calibrate the model to an existing epidemic curve.

Once calibration is done, we use data from SISSS, which records influenza-like-illness cases that are not confirmed by laboratory tests, for setting the initial simulation conditions of each urban area. This fact doesn’t affect the validity of our results because the purpose is to compare yearly/monthly numbers under different climate conditions rather than know the accurate number of infected individuals.

### Experimental setup

We have performed different tests to validate our approach and simulator. We first validated the simulator against influenza surveillance data, then we evaluated two different environmental scenarios. We believe that our simulator can be useful to predict the short- and medium-term spread of an infection, as well as to assess the effects that changes in climate can have over influenza epidemics worldwide.

The first scenario involves real climate values from AEMET and allows studying the short- and medium-term propagation for influenza strands. For the second set of scenarios we generate fictitious values of RH and T by scaling the real values. Our idea is to study the effects of the changing climate conditions on influenza propagation. Simulations occur across the 92 largest cities in Spain, which account for a population of 21,320,965 inhabitants. The time span is 7 months starting from the day identified as the onset date in our data - the 13th of December of 2010.

In our experiments we have used data from 92 weather stations from the national network, distributed across the country. Each weather station collects the values of temperature, atmospheric pressure, and relative humidity every 10 minutes during the entire 2011. These consists of about 157,000 data samples per station and 14.5 million data values in total. Based on these values, we generate the basic reproduction numbers to obtain an R0 value per urban area at every 10 minutes. With the previously determined initial influenza-like rates per region and (year-specific) date of onset, and after calibration, each urban region data - vaccination rates, individuals’ characteristics, initial infective individuals, and R0s values - are loaded from files.

## Results

### Validation of the simulator quality

The validation of our simulator in terms of its capacity to predict qualitatively similar propagation results as those approximated from the influenza surveillance data recorded by SISSS. The simulated values for each of the Spanish regions are the aggregated values of all the urban regions belonging to it.

**Fig. 3:**
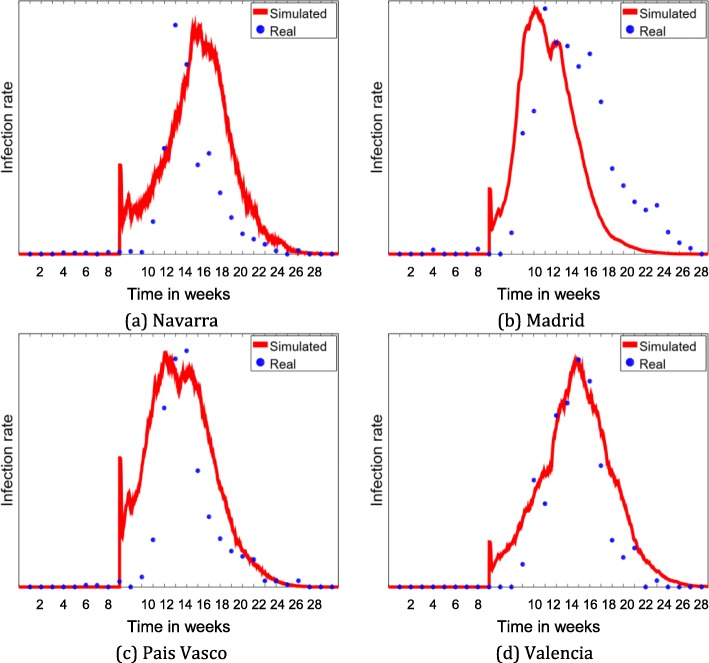
Comparison of normalized values between EpiGraph (in red) and real values (in blue) for Navarra, Madridm Pais Vasco and Valencia comunities. Real values correspond to the total number of infected Ntot, obtained by means Eq. 4 from influenza surveillance data

Figure 3 shows the simulated and actual estimated data. The simulated values are scaled to make the largest simulated value to be the same as the maximum real value. This allows a comparison of the evolution of the influenza propagation for each community over time. We can observe that although not perfect, the prediction shows a similar evolution with those from real scenarios. Note that the simulator considers an approximation of the real conditions during the simulated period, but producing a better (unlikely perfect) fit between the two domains would need to consider all the factors of the real world that affect the flu propagation at nation-level. Some of these are possibly unknown, others are not currently measured, and yet others are not possible to measure.

The reason for scaling the data is that the simulated and actual estimated data reflect the population rather differently. On one hand, the simulated values correspond to the overall number of individuals infected with influenza across the considered urban areas. These take into account all the individuals within the simulated areas but only include the largest urban regions (above 100,000 inhabitants); small cities, towns, and villages are not considered. On the other hand, the influenza surveillance data are only related to a small fraction of the existing clinical cases: SISSS covers a representative but small percentage of the population, in addition to the fact that there are more cases than those reported due to people not seeking medical attention. In contrast, the number of cases are collected from the complete community (including both large and small populations). It is thus not possible to compare the absolute values of the two data sources, although they should be linearly related.

### Effect of long-term climate changes

**Fig. 4:**
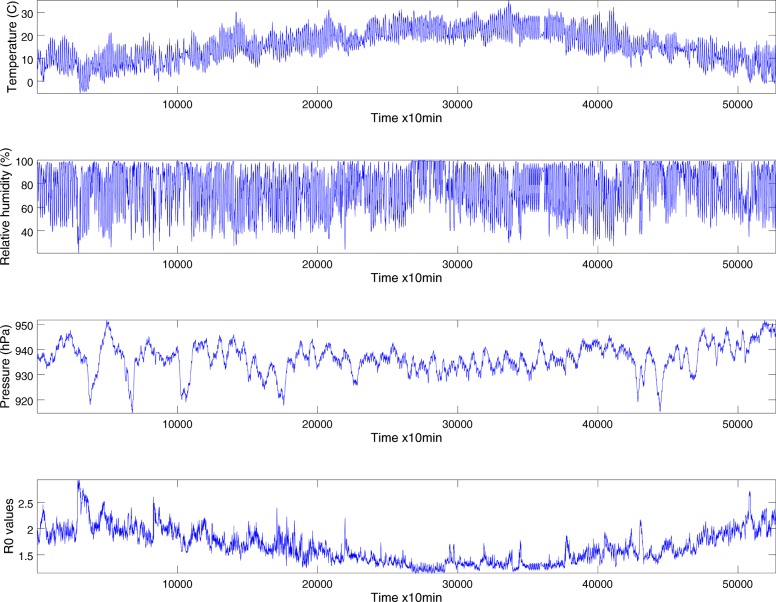
Meteorological parameters (T, RH, P) and the obtained R0s values for a one-year simulation (2011) for Tarrasa urban area

Figure 4 shows an example of the value of these parameters for the urban region of Terrasa (Barcelona) over one year. We can observe strong variations of R0 that are related to the changing temperature, relative humidity, and pressure conditions.

To evaluate the effect of both real and hypothetical meteorological climate changes on the spreading of influenza we evaluate temperature variations of *ΔT* degrees and percentage variations of the relative humidity *pRH*. *ΔT*=0 and *pRH*=1.0 correspond to the initial scenario with the original climate conditions. Studies show that climate change is producing increments in the average temperature (amplified by pollution) and, in southern Europe, longer periods of drought. The idea is to evaluate the impact of these changes on the influenza propagation. In this section, we consider long-term meteorological climate changes, that is, changes in the climate conditions that extend to the entire simulated period of 28 weeks. In this context, we evaluate two different scenarios, probably not as complex as future real climate changes.

**Fig. 5:**
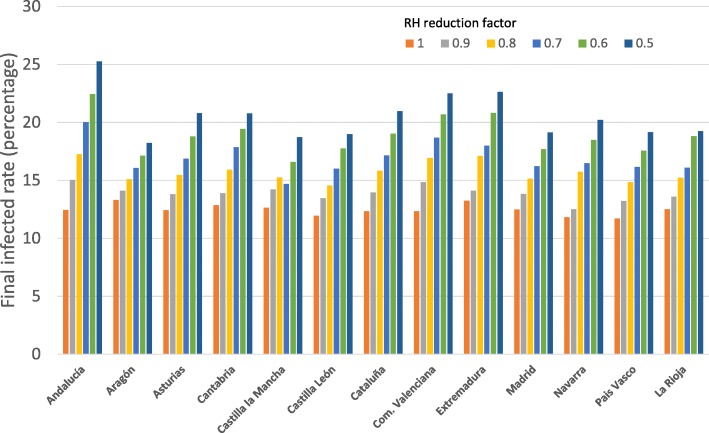
Effect of long-term changes of relative humidity on the influenza propagation for the different communities considered in the simulation

The first one corresponds to drought conditions, when the relative humidity values (RH) are smaller than current ones. We have considered a reduction of the relative humidity from 90% to 50% in increments of 10% (RH values half than the original ones). According to the infection model, influenza propagates easier for smaller RH values; we thus expect to observe a larger effect. Figure 5 shows the overall percentage of infected individuals per community predicted by EpiGraph. The diminishing RH has indeed a strong impact on the number of infected individuals. On average, 12.4% of the population was infected in the base case (reduction factor equal to 1), while the average infection rate for 0.5 factor is 20.5%. We can observe that a percental reduction of RH of 10% produces an approximate increment of 1.6% in the final infection rate.

**Fig. 6:**
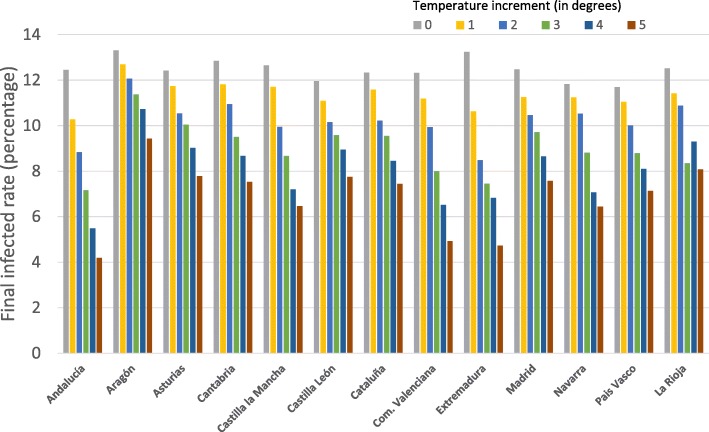
Effect of long-term changes in the temperature on the influenza propagation for the different communities considered in the simulation

The second scenario evaluates the impact of an increase of temperature on the propagation. Figure 6 shows the final infection rate for an increment of the temperature between 0 degrees (current case) and 5 degrees Celsius. We can observe that now there is a reduction in the infection rate when the temperature increases. Now, an increment of 5 degrees reduces the average infection rate from 12.5% to 6.9%—a decrement of 1.1% per degree.

Both scenarios assume that the values of the parameters (RH, T) change one at a time. This is a simplification, and the idea behind this approach is to evaluate the impact of a single parameter variation on the overall influenza outcome. However, EpiGraph supports specifying any changing combination of climate conditions. In a more realistic scenario both parameters would change, and the climate specialists are those who should define what the concrete values are.

**Fig. 7:**
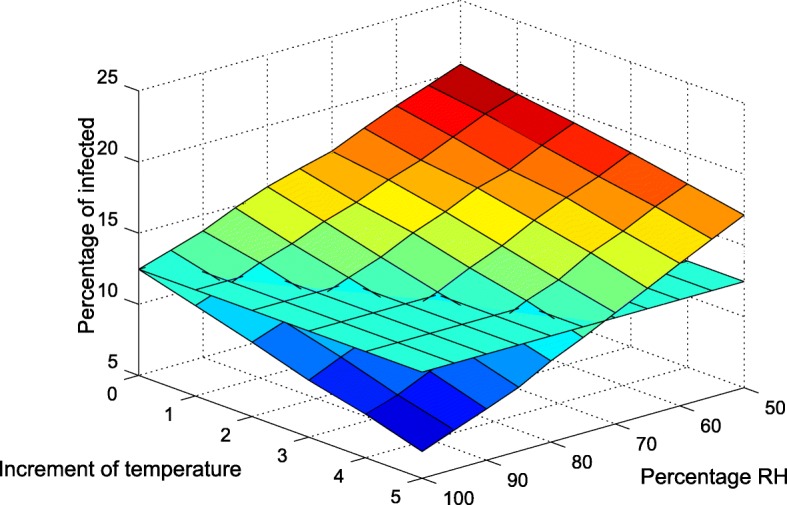
Effect of long-term changes in the relative humidity (percentil reduction) and temperature (value increment in Celsius) on the influenza propagation for the average nation-wide infection rates

Figure 7 shows the combined effect of temperature and relative humidity change on the average nation-wide infection rate. We have plotted two planes: the first one (colored) represents the average infection rates for different increments in the temperature values and percentile reductions in the relative humidity; the second one (green) displays the infection rate of the original scenario (without climate variation) for all the coordinates and represents the baseline case. The two planes intersect in the lower-left point, where the temperature and RH have the original values. Although both parameters influence the final infection rate, relative humidity has a larger effect than temperature.

**Fig. 8:**
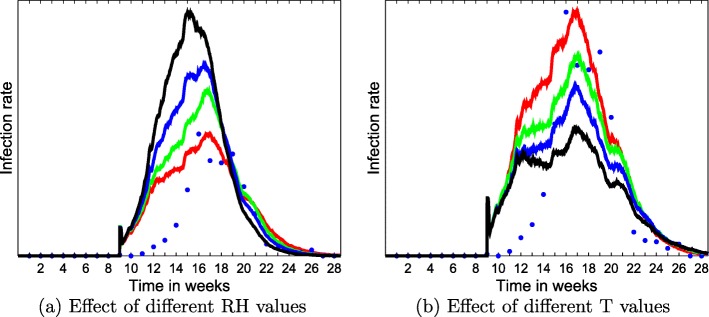
Effect of long-term parameter variation on the infection distribution shape for Andalucía. In **a** different RH scales are evaluated (100% in red, 90% in green, 80% in blue and 70% in black); In **b** different temperature offsets are evaluated (0 degrees in red, +1 degrees in green, +2 degrees in blue and +3 degrees in black)

Figure 8 shows the effect of RH and temperature variations on the infection distribution for Andalucia community. We can observe that the variation of both parameters changes the shape of the distribution, especially in terms of the peak values but also - more subtly - in terms of the propagation interval.

The maximum and minimum 95% confidence intervals baseline scenario (no RH reduction nor temperature increment) ranges between 0.28 and 0.06 for urban areas in Castilla la Mancha and Aragón, respectively. These results are produced by a simulator repeating the simulations 30 times. Note that there already exist uncertainty in the input data, both with respect to the number of initially infected individuals as well as from the point of view of the epidemic model.

### Effect of short-term climate changes

**Fig. 9:**
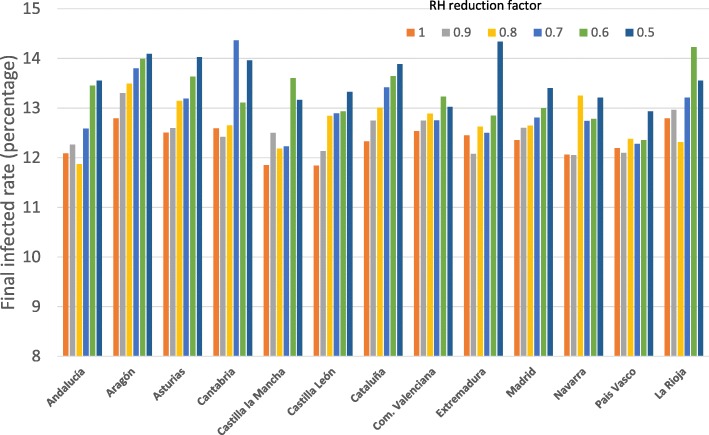
Effect of short-term changes in the relative humidity on the influenza propagation for the different communities considered in the simulation: in color the average infection rates for different increments in the temperature values and percentile reductions in the relative humidity; in green the infection rate of the scenario without climate variation

**Fig. 10:**
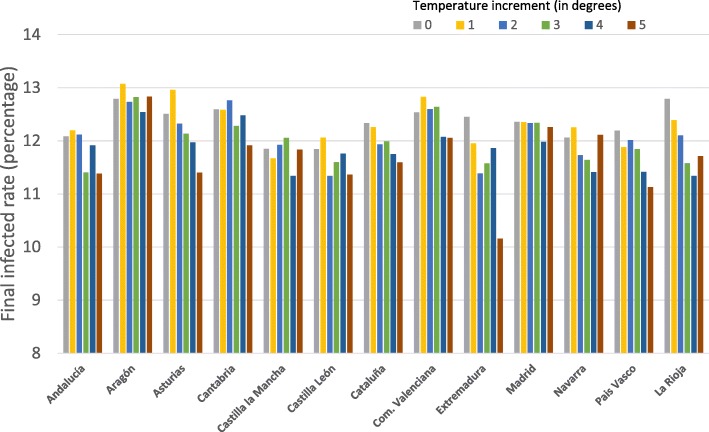
Effect of short-term changes in the temperature on the influenza propagation for the different communities considered in the simulation

To evaluate the effect of short-term changes in climate conditions, we modify RH and the temperature exactly like described in the previous section, only for the first week of the simulation. The rest of the simulation uses the original climate parameters. Figures 9 and 10 show the final infection rate for different variations of RH and temperature. We can observe that the impact on the overall percentage of infected individuals is still important, particularly for a decrease in *RH* of 0.2 or more and - less evidently - for an increase in temperature of 3 degrees or more. For smaller changes in temperature the effect is less evident, but we believe that this is due to the fact that the short-term simulation of temperature increase is only one week.

## Discussion

We achieve herd immunity in two ways: as result of vaccinating campaigns, and naturally when an individual that was infected goes to the recovery (or dead) state, in which case he becomes immune and starts acting as a propagation stopper. As a result, after certain threshold of infected vs susceptible individuals, the infection rate naturally goes down. This occurs at the inflection point in the propagation graph, specifically at about 16 weeks (in our data). The vaccination success rate during the 2010-2011 season was approximately 50% [39]. Given the parameters shown in Fig. 4 of [40] for herd immunity for influenza, we consider that the *R*0 considered by our model takes into consideration this type of immunity. We do not model the level at which herd immunity starts acting as a parameter, although this phenomenon occurs naturally in the simulations. The simulator is flexible enough to support different daily contact patterns for each individual. The probability of an individual getting infected during an interaction also differs (it’s a stochastic process), and thus the infection can be transmitted to individuals pertaining to different groups.

Recently, the work in [41] suggests that RH should also be considered (together with the temperature) as a modulating factor in the influenza propagation. Another study that analyses this relationship can be found in [42]. This work provides a transmission risk contour map based on the temperature and RH humidity values. Note that our work addresses the problem of evaluating the influenza propagation from a different perspective. Instead of analyzing the propagation mechanisms of the virus and how they are related to the environment conditions, we focus on an empirical relationship between the virus’s basic reproduction number and the outdoor specific humidity. The R0 values used in this work are the combination of both outdoor and indoor virus propagations, and provide an approximation of a real scenario. Note also that the main goal of this work is to evaluate the impact of the weather conditions on the propagation.

A possible limitation is that we only model the largest 92 urban regions in Spain; we could add more information related to smaller cities and towns, including rural regions. Nevertheless we don’t think this data would make a significant difference in the results, as the infection needs a large number of hosts to explode, and / or travel patterns between the infected areas. Small town and village areas are arguably much less likely than cities to fulfill these roles.

A second limitation is related to the meteorological factors affecting the infection propagation: the number and set of climate factors that the meteorological model takes into account, and the choice of the model itself. Additional parameters that specialists mention as possible influencers in virus transmission are factors such as wind, precipitation, or pollution.

One important thing to underline is that the data that the study [5] (whose model we adopt) is based on is of real cases and spans 30 years. Interactions between meteorological trends and human behavior are therefore intrinsically reflected in the data, although the rules of behavior change are not explicitly specified for the agents (i.e. individuals) involved in the simulation. The case can be made that meteorological changes were not as extreme before 2002, and that a regression model based on new data may change as well over time. While this is a definite possibility, we believe that its nature will not change in a fundamental way, such that we can still predict trends, if not absolute values.

A third limitation is that we don’t calibrate the model on an epidemic curve, which results in different timings of the flu peaks in some regions, such as in Navarra and Madrid.

Finally, to successfully simulate the flu epidemics requires leveraging many different types of data, most of them in large amounts, as input and calibration measurements for our tool (EpiGraph). For instance, we are using social network data from Enron and Facebook to set up the population interaction patterns, census data to extract the characteristics of the different types of individuals, Google Maps to initialize the transportation module, data from AEMET to run simulations that are realistic from a meteorological viewpoint, and weekly ILI rates obtained from the SISSS to initialize and evaluate the simulator. This makes the implementation of EpiGraph more realistic, a strength that can lead to more accurate simulations.

## Conclusions

We have extended our simulator EpiGraph with a meteorological model that interacts with the rest of the system to better reflect the behavior of the influenza propagation through the entire population of Spain. To produce realistic results we also take into account vaccination, with different ratios based on the individuals’ ages. The simulator results are compared to real data on infection rates and across the whole country. The results for the prediction of the evolution of the influenza propagation for each community over time are similar in shape to the real data. After validating the simulator, we evaluate different scenarios that reflect changes in climate conditions, and show the predictions for variations in the relative humidity and temperature. Lastly, we make EpiGraph‘s source code publicly available at [18], to be used by the scientific community.

As future work, an interesting, although independent, possibility is to investigate the potential of EpiGraph to simulate the evolution of the virus spread for different subtypes of influenza, once the propagation model parameters (e.g. incubating period, infectious period, basic reproduction numbers, etc.) are known, or to narrow down the possible subtypes in early phases of an infection. One could also investigate the impact of new meteorological factors on the evolution of the infection.

## Data Availability

EpiGraph’s User Manual and source code are publicly available at [18] and can be used by the scientific community. The dataset supporting the conclusions of this article is available in the [43] repository.

## Abbreviations

SISSS: Influenza Sentinel Surveillance System
AEMET: Spanish Meteorological Agency
*MM*: EpiGraph’
s meteorological model; SIR/SIRS: Susceptible Infected Recovered / - Susceptible
*SH*: specific humidity
*RH*: relative humidity
*T*: temperature
*P*: atmospheric pressure
ILI: influenza-like illness
ISCIII: Institute of Health Carlos III of Madrid
